# Modulation of human V*α*24^+^V*β*11^+^ NKT cells by age, malignancy and conventional anticancer therapies

**DOI:** 10.1038/sj.bjc.6602218

**Published:** 2004-11-02

**Authors:** T Crough, D M Purdie, M Okai, A Maksoud, M Nieda, A J Nicol

**Affiliations:** 1 Department of Medicine, University of Queensland, Brisbane, Australia; 2 The Queensland Institute of Medial Research, Brisbane, Australia; 3 Bone Marrow Transplant Unit, Royal Brisbane Hospital, Brisbane, Australia; 4 Yokohama City University School of Medicine, Japan

**Keywords:** NKT cells, immune therapy, age, human

## Abstract

Immunotherapy strategies aimed at increasing human V*α*24^+^V*β*11^+^ natural killer T (NKT) cell numbers are currently a major focus. To provide further information towards the goal of NKT cell-based immunotherapy, we assessed the effects of age, cancer status and prior anticancer treatment on NKT cell numbers and their expansion capacity following *α*-galactosylceramide (*α*-GalCer) stimulation. The percentage and absolute number of peripheral blood NKT cells was assessed in 40 healthy donors and 109 solid cancer patients (colorectal (*n*=33), breast (*n*=10), melanoma (*n*=17), lung (*n*=8), renal cell carcinoma (*n*=10), other cancers (*n*=31)). Responsiveness to *α*-GalCer stimulation was also assessed in 28 of the cancer patients and 37 of the healthy donors. Natural killer T cell numbers were significantly reduced in melanoma and breast cancer patients. While NKT numbers decreased with age in healthy donors, NKT cells were decreased in these cancer subgroups despite age and sex adjustments. Prior radiation treatment was shown to contribute to the observed reduction in melanoma patients. Although cancer patient NKT cells were significantly less responsive to *α*-GalCer stimulation, they remained capable of substantial expansion. Natural killer T cells are therefore modulated by age, malignancy and prior anticancer treatment; however, cancer patient NKT cells remain capable of responding to *α*-GalCer-based immenotherapies.

Natural killer T (NKT) cells are a unique lymphocyte subset, which in humans are characterized by the presence of an invariant T-cell receptor (TCR) V*α*24^+^V*β*11^+^ associated with the natural killer (NK) receptor NKR-P1A (Dellabona *et al*, 1994). Unlike conventional T cells human NKT cells and their murine counterpart, V*α*14 NKT cells are activated by the glycolipid, *α*-galactosylceramide (*α*-GalCer) presented by a nonpolymorphic major histocompatability complex (MHC) class I-like molecule CD1d (Kawano *et al*, 1999b; Nieda *et al*, 1999). Natural killer T cells have been shown to have strong antitumour activity upon *α*-GalCer stimulation (Kawano *et al*, 1998; Nicol *et al*, 2000b; Kikuchi *et al*, 2001; Nieda *et al*, 2001). As a consequence, clinical trials aimed at activating and increasing NKT cell numbers are currently a major focus for immune therapy of cancer.

There are limited studies detailing the normal range of human peripheral blood (PB) NKT cells numbers and the effects of parameters such as age, malignant disease and associated treatment on their number and function. As the ageing process and immune dysfunction due to malignant disease have been shown to impact other cell types, these factors require consideration for NKT cells (Goto *et al*, 1999; Ninomiya *et al*, 1999; Hakim *et al*, 2004; Plackett *et al*, 2004).

The effect of the ageing process on NKT cell numbers has only been assessed in a single study, where the percentage of V*α*24^+^ T cells was shown to decrease with age (DelaRosa *et al*, 2002). The impact of age, however, on NKT cell expansion following *α*-GalCer stimulation has not yet been evaluated (DelaRosa *et al*, 2002). If NKT cells do contribute to the normal protective mechanisms against cancer, a reduction in NKT cell number or function as a result of the ageing process age may contribute to the higher incidence of cancer in this group of patients.

The effects of malignancy on NKT cell number and function remain unclear. Significant reductions in PB NKT cell numbers have been reported in patients with melanoma, prostate cancer and lung cancer (Kawano *et al*, 1999a; Tahir *et al*, 2001; Motohashi *et al*, 2002), however in other studies the numbers of NKT cells in advanced cancer patients are comparable to those in healthy individuals (Yanagisawa *et al*, 2002). Variations in NKT cell function have also been described. Decreased responsiveness of NKT cells to *α*-galactosylceramide (*α*-GalCer) stimulation has been demonstrated in prostate cancer patients and advanced cancer patients (Tahir *et al*, 2001); however, in contrast NKT cells of lung cancer and melanoma patients have been shown to have comparatively normal *α*-GalCer proliferative capacity (Kawano *et al*, 1999a; Motohashi *et al*, 2002).

Of note, previous studies investigated NKT cells of cancer patient's either pretreatment or did not take into account potential effects of chemotherapy and radiation therapy on NKT cell numbers and expansion capacity. This is important information due to the likelihood that NKT cell immunotherapy will be used secondary to conventional cancer therapies.

For interventions aimed at increasing NKT cells to be effective at treating malignancy, it is a prerequisite that NKT cells in cancer patients firstly exist and also retain the ability to respond to specific stimuli. This study evaluated the effects of age, malignancy and associated treatment on the numbers of NKT cells in 109 patients with solid organ cancer using 40 healthy individuals as controls. Responsiveness to *α*-GalCer stimulation was also assessed in 28 of the cancer patients and 37 of the healthy donors following assessment of NKT cell numbers.

## MATERIALS AND METHODS

### Subject groups and controls

The study group consisted of 109 subjects (64 males and 45 females) with solid organ cancer (median age 58 years, range 26–82). They were subclassified according to cancer type (colorectal, breast, melanoma, renal cell carcinoma (RCC), lung, others) and prior cancer treatment. The control healthy volunteer group comprised 40 healthy volunteers, 25 females and 15 males (median age 34 years, range 19–68) with no history of malignant disease. Natural killer T cell number was assessed in each of the cancer patients and healthy donor, however only 37 of the healthy donors and 28 of the cancer patients could be assessed for expansion capacity following *α*-GalCer stimulation.

Characteristics of the study group are presented in Table 1

**Table 1:** Characteristics of each group of cancer subjects and normal donors

				**Sex**		**Treatment history**			
**Subject group**	***n***	**Age range**	**Median age**	**Males**	**Females**	**No prior treatment**	**Prior chemotherapy**	**Prior radiation**	**Prior chemotherapy and Radiation**
Colorectal	33	43–79	61	22	11	13	18	0	3
Breast	10	41–69	54	0	10	1	2	0	7
Other cancers	31	27–72	58	13	18	13	16	1	2
Melanoma	17	26–83	59	14	3	13	1	3	1
Lung cancer	8	33–72	55	6	2	2	2	2	2
RCC	10	36–67	53	9	1	9	0	1	0
All cancer cases	55	26–83	58	64	45	50	39	7	15
Healthy donors	40	19–68	34	15	25	40	NA	NA	NA

NA=not applicable.

. Peripheral blood samples were collected after obtaining informed consent from the subjects, and the study was approved by The Royal Brisbane and Womens Hospital and the Health Services Districts Human Research Ethics Committee.

### Phenotypic analysis

Fresh whole blood or peripheral blood mononuclear cells (PBMC), separated by Ficoll–Paque (Amersham Pharmacia Biotech, Uppsala, Sweden) density centrifugation were stained with fluorescein isothiocyanate (FITC)-conjugated anti-V*α*24 (IgG1: Cl5), phycoerythrin (PE)-conjugated anti-V*β*11 (IgG2a: C21) and phycoerythrin–cyanin 5.1 (PC5)-conjugated anti-CD3 (IgG1, UCHT1) and analysed by three-colour flow cytometry using a FACSCalibur (Becton Dickinson, San Diego CA, USA). Appropriate isotype controls were used and all antibodies were purchased from Beckman Coulter (Sydney, Australia). Natural killer T cells were defined as V*α*24^+^V*β*11^+^CD3^+^ and to ensure accuracy of the flow cytometric evaluation up to 1 × 10^6^ cells were assessed in order to acquire >100 NKT cell events.

### Calculation of NKT cell numbers

Natural killer T cell numbers were expressed as a percentage of the CD3^+^ lymphocyte population as determined by flow cytometry. To determined the number of NKT cells per litre (l) of blood, the fraction of lymphocytes that were NKT cells according to flow cytometry was multiplied by the number of lymphocytes per litre as determined by an automated full blood count on the same sample.

### Expansion of V*α*24^+^V*β*11^+^ NKT cells

Peripheral blood mononuclear cells were assessed for NKT cell numbers as described above (Day 0) and cultured at 1 × 10^6^ cells ml^−1^ in AIM-V medium (Gibco BRL, Melbourne, Australia) supplemented with 10% normal human AB serum, 10Uml^−1^ interleukin-2 (IL-2) (R&D Systems, Sydney, Australia) and 100 ngml^−1^ KRN 7000 (a gift from Pharmaceutical Division, Kirin Brewery, Gumna, Japan) for 7 days. On Day 7, the cells were harvested, counted and analysed by flow cytometry as described above and the results are expressed in terms of fold expansion, as determined from the respective NKT numbers at Day 0 and following 7 days expansion.

### Statistics

Results for NKT cell percentages and absolute numbers of NKT cells and CD3^+^ T cells have been presented as medians separately for the different cancer groups. Owing to the large number of zero NKT cell percentage counts, the distribution was extremely skewed to the right and thus standard normal distribution-based statistics were not appropriate. For crude analysis, Kruskal–Wallis nonparametric analysis of variance was used to compare median NKT cell percentages and NKT l^−1^ between cancer groups. Two-way comparisons between the various cancer groups and healthy volunteers were performed using Mann–Whitney *U*-tests. Both NKT cell percentage and absolute NKT cell numbers followed an approximately negative-binomial distribution, and thus negative-binomial regression was used to evaluate the statistical significance of its association with tumour status, after adjusting for age and sex differences. The degree of association between age and NKT cell percentage or NKT l^−1^, age and fold expansion and NKT percentage and fold expansion were calculated using a Spearman's nonparametric correlation coefficient.

## RESULTS

### Effect of malignancy on PB NKT cell numbers

Natural killer T cells as a percentage of CD3^+^ T cells (NKT %) and the absolute number of NKT cells l^−1^ of PB were assessed in healthy donors (*n*=40) and cancer patients (*n*=109) with a broad range of malignancies. Natural killer T cells comprised up to 1.27% of CD3^+^ T cells (median value 0.113%) and 9.89 × 10^6^ l^−1^) of PB in healthy donors, and up to 1.19% of CD3^+^ T cells (median value 0.02%) and 6.52 × 10^6^ l^−1^ (median value 0.2 × 10^6^ l^−1^) of PB in all cancer patients combined (Table 2

**Table 2:** NKT cell percentages, absolute number of NKT cells l^−1^ (× 10^6^) and absolute number of CD3^+^ T cells l^−1^ (× 10^9^) of PB for each study group in comparison to healthy volunteers

		**NKT %**			**NKT l−1 (× 10^6^)**			**CD3^+^ T cells l^−1^ (× 10^9^)**		
**Subject group**	***n***	**Range**	**Median**	*P*-value*	**Range**	**Median**	*P*-value*	**Range**	**Median**	*P*-value*
Colorectal	33	0–0.39	0.012	0.17	0.00–6.53	0.17	0.15	0.19–1.87	1.08	0.06
Breast	10	0–0.15	0.01	0.02	0.00–3.32	0.12	0.13	0.25–2.10	1.00	0.01
Other cancers^a^	31	0–1.19	0.04	0.86	0.00–3.24	0.31	0.20	0.16–2.24	0.90	0.003
Melanoma	17	0–0.17	0.01	0.04	0.00–1.61	0.17	0.01	0.25–2.04	0.69	0.004
Lung cancer	8	0–0.26	0.03	0.61	0.00–2.10	0.25	0.09	0.33–1.41	0.85	0.01
RCC	10	0–0.77	0.04	0.66	0.00–1.60	0.52	0.82	0.49–1.74	1.04	0.25
All cancer cases	109	0–1.19	0.02	0.28	0.00–6.53	0.20	0.06	0.16–2.24	0.92	<0.001
Healthy Donors	40	0–1.27	0.11	Reference	0.00–9.89	1.93	NA	0.66–4.52	1.43	Reference

NKT cell percentage is calculated as a percentage of the total CD3^+^ T cells as determined by flow cytometry.

* *P*-values compared with healthy donors obtained from negative binomial regression models adjusted for age and sex differences between study groups.

a Other cancers include liver, adenocarcinoma, prostate, ovarian, uterine and stomach cancer.

NA=not applicable.

). No significant difference was observed between the frequency (*P*=0.28) or number (*P*=0.06) of circulating NKT cells in cancer patients and those in healthy donors after adjusting for age and sex differences. Analysis of the individual cancer subgroups showed melanoma patients (*n*=17) to have a significantly lower NKT% (median 0.01%, *P*=0.04) and NKT l^−1^ (median 0.17 × 10^6^ l^−1^, *P*=0.01), and breast cancer patients (*n*=10) to have a reduced frequency of NKT cells (median 0.01, *P*=0.02) but comparatively similar absolute numbers of NKT cells (*P*=0.13). Total CD3^+^ T cells l^−1^ of PB were significantly lower in all cancer groups with the exception of colorectal cancer and RCC, indicating that the reduced NKT% observed in melanoma and breast cancer patients could not be attributed to increased numbers of total PB T cells.

### Impact of prior radiation and/or chemotherapy treatment on NKT cell numbers

Statistical comparison was conducted between the NKT cell numbers of all cancer patients who had not undergone previous chemotherapy or radiation treatment and those who had not undergone each treatment respectively. No significant difference was detected between the groups in either comparison, suggesting that neither radiation nor chemotherapy had an independent effect on NKT cell numbers in cancer patients when all cancers were considered collectively (Table 3

**Table 3:** Effects of prior radiation and chemotherapy treatment on NKT cell percentages and absolute numbers of NKT cells and CD3^+^ T cells in cancer patients

		**NKT %**		**NKT l^−1^ (× 10^6^)**		**CD3 l^−1^ (× 10^9^)**	
**Subject group**	***n***	**Median**	*P*-value*	**Median**	*P*-value*	**Median**	*P*-value*
Prior radiation treatment	22	0.02	0.95	0.14	0.28	0.82	0.10
No prior radiation treatment	87	0.02		0.24		1.00	
							
Prior chemotherapy treatment	54	0.02	0.26	0.21	0.26	1.04	0.83
No prior chemotherapy treatment	55	0.03		0.20		0.87	

* *P*-value obtained from negative binomial models adjusting for age and sex differences between the groups.

).

To assess whether chemotherapy or radiation treatment modulated NKT cell numbers in the individual cancer subgroups, statistical analysis was conducted for each group following exclusion of these patients who had previous chemotherapy (Table 4

**Table 4:** NKT cell percentages, absolute number of NKT cells l^−1^ (× 10^6^) and absolute number of CD3^+^ T cells l^−1^ (× 10^9^) of PB for each study group excluding subjects who have had prior chemotherapy in comparison to healthy volunteers

**Subject group**	***n***	**NKT %**	*P*-value*	**NKT l^−1^ (× 10^6^)**	*P-*value*	**CD3^+^ T cells l^−1^ (× 10^9^)**	*P*-value*
Colorectal	12	0.016	0.09	0.14	0.22	0.87	0.01
Breast	1	0.000	NA	0.00	NA	0.00	NA
Other cancers^a^	13	0.130	0.27	1.33	0.72	1.03	0.06
Melanoma	15	0.010	0.03	0.08	0.002	0.66	<0.001
Lung cancer	4	0.015	0.01	0.17	0.009	0.82	0.03
RCC	10	0.040	0.62	0.52	0.96	1.04	0.09
All cancer cases	55	0.030	0.83	0.20	0.20	0.87	<0.001
Healthy donors	40	0.113	Reference	1.93	NA	1.43	Reference

See footnote in Table 2.

) or previous radiation therapy (Table 5

**Table 5:** NKT cell percentages, absolute number of NKT cells l^−1^ (× 10^6^) and absolute number of CD3^+^ T cells l^−1^ (× 10^9^) of PB for each study group excluding subjects who have had prior radiation therapy in comparison to healthy volunteers

**Subject group**	***n***	**NKT %**	*P-*value*	**NKT l^−1^ (× 10^6^)**	*P*-value*	**CD3^+^ T cells l^−1^ (× 10^9^)**	*P*-value*
Colorectal	30	0.016	0.16	0.17	0.14	0.10	0.03
Breast	3	0.005	0.001	0.01	0.02	0.65	0.001
Other cancers^a^	28	0.042	0.91	0.32	0.15	1.01	0.003
Melanoma	13	0.018	0.10	0.17	0.02	0.70	0.013
Lung cancer	4	0.055	0.92	0.25	0.12	1.11	0.19
RCC	9	0.04	0.31	0.38	0.12	1.03	0.06
All cancer cases	87	0.02	0.28	0.24	0.24	1.00	0.001
Healthy donors	40	0.113	Reference	1.93	Reference	1.43	Reference

See footnote in Table 2.

). As shown in Table 4, the frequency and absolute number of NKT cell numbers remained significantly lower in melanoma patients, indicating chemotherapy was not responsible for the reduction in NKT cells. However, it must be noted that this result is not unexpected as chemotherapy is not a common treatment modality for this cancer type. Conversely, following exclusion of those melanoma patients with a history of radiation treatment (Table 5), NKT cell numbers in the group were returned to comparatively healthy levels. This result suggests that radiation therapy may have a direct effect on NKT cell numbers in patients with melanoma.

In the instance of breast cancer, there were insufficient patients in this subgroup following exclusion to ascertain whether prior treatment was responsible for the decreased NKT % previously observed. Furthermore, while the significant difference detected between healthy donors and lung cancer patients with no history of chemotherapy treatment (*P*=0.01) may suggest chemotherapy-mediated modulation of NKT cell numbers; the low subject numbers (*n*=4) in this subgroup does not facilitate accurate analysis.

### Effect of the ageing process on NKT cell numbers

**Figure 1:**
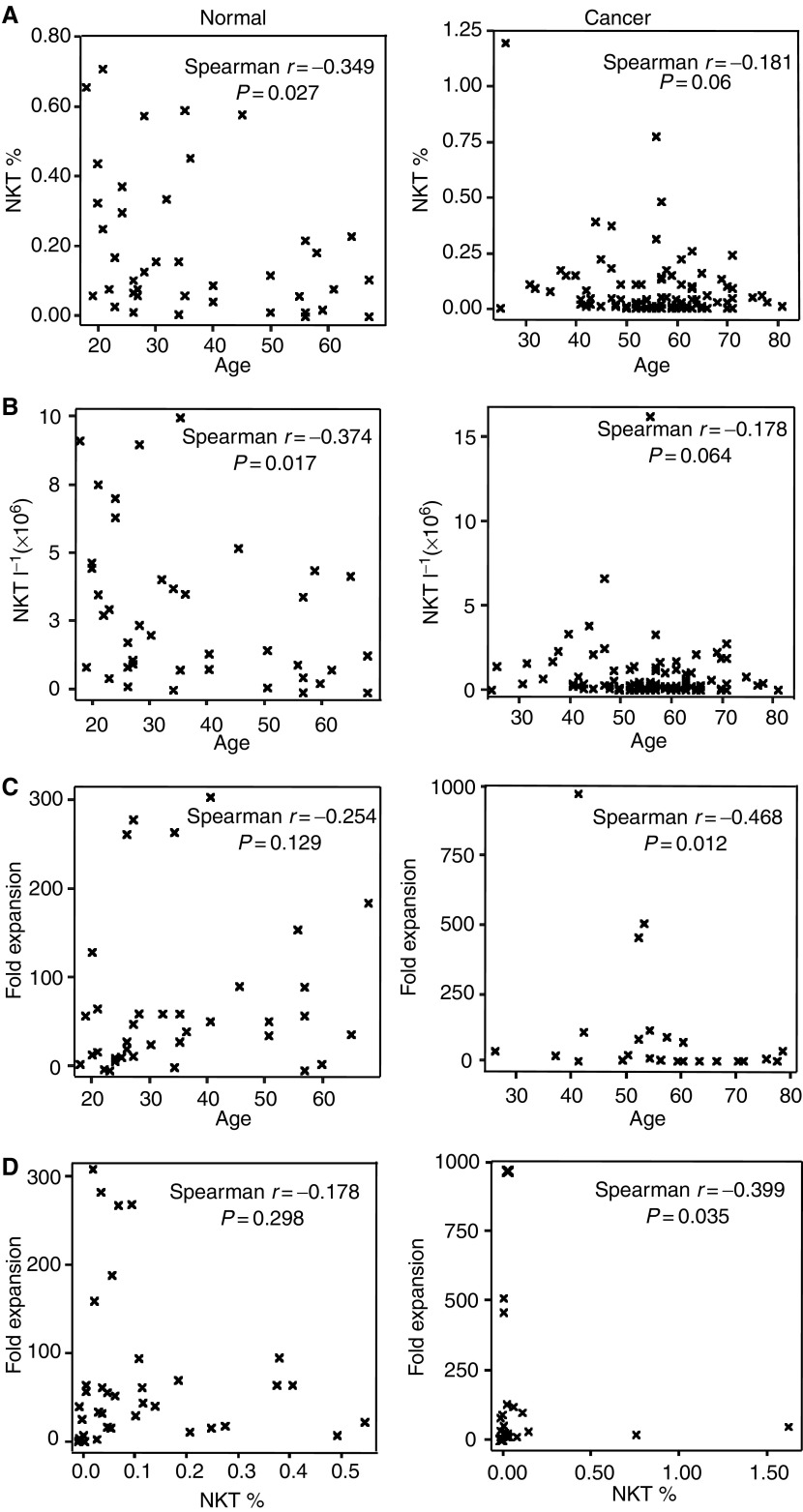
Association of age on proportion of NKT cells (NKT %; **A**), absolute numbers of circulating NKT cells (NKT l^−1^; **B**) and responsiveness to *α*-GalCer (**C**) in cancer patients and healthy donors. (**D**) represents the correlation between NKT cell numbers and NKT cell expansion capacity.

The percentage and absolute number of circulating NKT cells in healthy donors weakly but significantly correlated with age such that NKT % and NKT l^−1^ decreased with increasing age (Figure 1

, panels A and B). While the number of NKT as a percentage and per litre of blood of cancer patients also appeared to decrease with increasing age, this correlation proved insignificant (Figure 1, panels A and B).

### Expansion capability of NKT cells in cancer patients and healthy volunteers – the effect of age and malignancy

**Figure 2:**
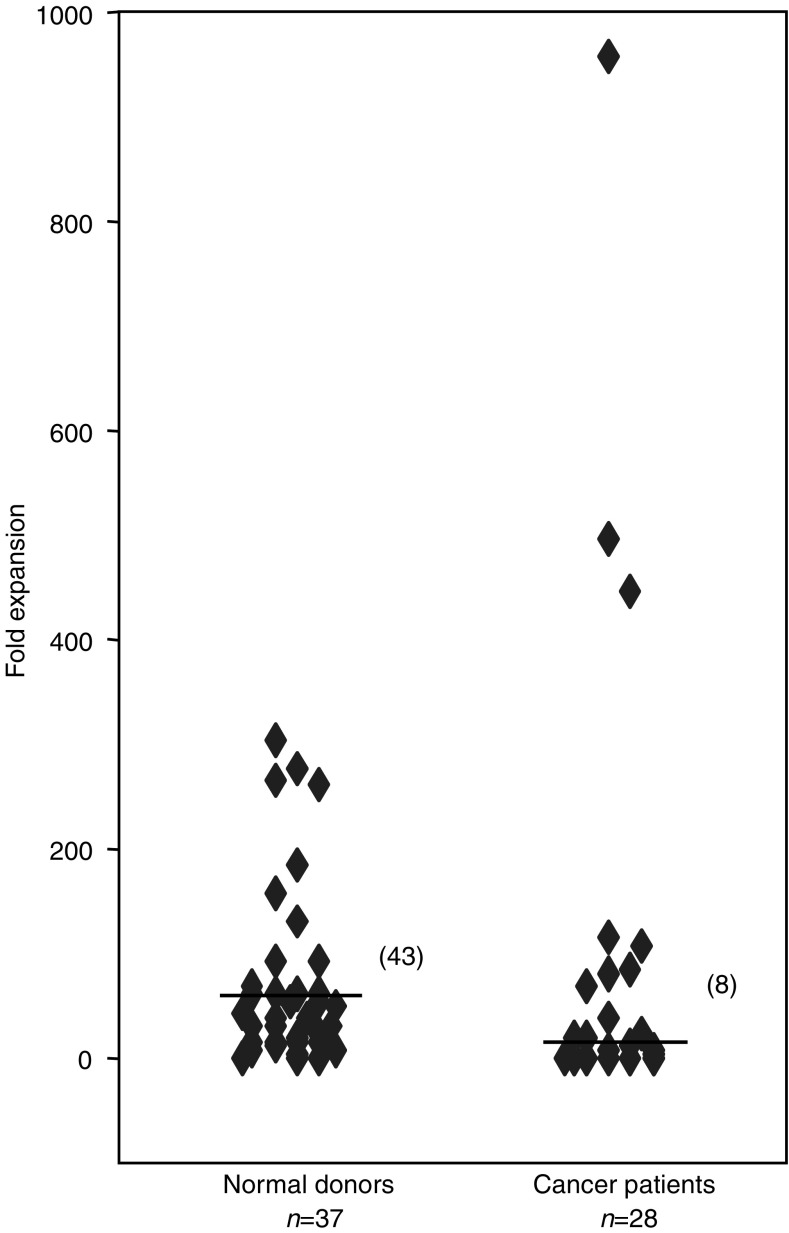
Expansion capacity of NKT cells in normal donors (*n*=37) and cancer patients (*n*=28) in response to *α*-GalCer stimulation (line indicates median of group). Natural killer T cells can rapidly expand in both groups, however overall to a significantly lower degree in cancer patients (*P*=0.02).

Stimulation by *α*-GalCer resulted in the rapid expansion of NKT cells from most cancer patients and healthy donors. Figure 2

displays the expansion capacity (fold expansion) of healthy donor (*n*=37) and cancer patient (*n*=28) NKT cells in response to *in vitro α*-GalCer stimulation. Natural killer T cells of healthy donors expanded between 0- and 303-fold and those of cancer patients expanded between 0- and 959-fold. While NKT cells of cancer patients had an overall reduced capacity to expand (median eight-fold) as compared to healthy donors (median 43-fold, *P*=0.02), some individual cancer patients were clearly better NKT cell expanders than healthy donors.

Contrary to the effects of age on NKT numbers in healthy donors, age did not significantly influence their fold expansion as displayed in Figure 1C. Although the proliferative potential of cancer patient NKT cells to *α*-GalCer appears to decrease significantly with age, conclusive analysis requires the assessment of increased numbers of younger cancer patients.

Although NKT cells expanded rapidly from most donors, there was no relationship between the NKT % in healthy donors and the degree to which the NKT cells expand. The results do suggest, however, that NKT % is a reliable indicator of fold expansion in cancer patients, although the highly skewed nature of the population towards low NKT % may make the spread of NKT % too low to determine the correlation accurately. It is of interest that the largest *in vitro* NKT cell expansion was observed from donors with relatively low NKT cell numbers.

## DISCUSSION

Clinical application of NKT cells as therapy for malignancy is critically dependent upon their activation and subsequent expansion in humans. With the development of therapeutic approaches with the potential to activate NKT cells *in vivo* (Nieda *et al*, 2003) and expand NKT cells *in vitro* (Nieda *et al*, 1999; Nicol *et al*, 2000a; Takahashi *et al*, 2000), it is important to more clearly define their applicability in the clinical setting. Previous data indicate that NKT cells may be decreased in some cancer patients (Kawano *et al*, 1999a; Tahir *et al*, 2001; Motohashi *et al*, 2002). It this is associated with or caused by decreased responsiveness to *α*-GalCer, then these therapies may have less than expected clinical utilities.

We have confirmed with considerably larger subject numbers in this study that the percentage of V*α*24^+^ NKT cells in cancer patients does not significantly differ to that in healthy donors when all cancer types are considered collectively (Yanagisawa *et al*, 2002). Our data also support previous reports that NKT cell numbers are significantly reduced in patients with melanoma (Kawano *et al*, 1999a); however, in contrast to Motohashi *et al* (2002) decreased frequency and absolute number of NKT cells was not seen in lung cancer patients. A likely explanation for this inconsistency is the inclusion of insufficient patients in this subgroup to detect a statistically significant difference, in view of the 60 lung cancer patients assessed in the previous study. As no studies to date have assessed the numbers of NKT cells in breast cancer patients we have, for the first time, demonstrated a significantly reduced percentage of NKT cells in this cancer group.

Reasons for the observed reductions in breast cancer and melanoma patients are unknown. It is evident that the results are not simply a consequence of increased total CD3^+^ T cell numbers in either disease (Table 2). Contrary to previous studies, which have in majority assessed NKT cell levels pretreatment or without indicating prior treatment, we have taken into consideration potential effects of chemotherapy or radiation treatment on NKT cell numbers. We have identified that radiation treatment may be directly responsible for the decreased NKT cell numbers in melanoma patients. It remains possible, however, that a disease-specific effect by melanoma may still contribute to the observed reduction and although not examined in this study, decreases in NKT cell numbers could also be due to an accumulation of NKT cells at tumour sites, thus decreasing their overall number in the circulating PB. This, together with longitudinal studies, aimed at ascertaining whether the low NKT cells numbers in these patients predated the manifestation of cancer, would help further the understanding of the relationship between NKT cell numbers and cancer.

There were insufficient subject numbers to ascertain treatment-related effects on NKT cells breast cancer patients, and indications that chemotherapy modulates NKT cell numbers in lung cancer patients cannot be conclusively determined with the subject numbers available in this subgroup.

The effect of the ageing process on NKT cell numbers has only been assessed in a singly study (DelaRosa *et al*, 2002). In accordance with that study, we showed a decreased percentage of V*α*24^+^ NKT cells in healthy elderly individuals. We have also demonstrated that absolute NKT cell numbers decrease significantly with age in healthy donors. A similar trend was observed in cancer patients, however the statistical significance (*P*=0.06) was potentially influenced by a concentration of middle- to late-aged cancer patients. Although greater numbers of younger cancer patients are required to assess this association accurately, the results do raise the possibility that low NKT cells may be a risk factor for cancer in young adults. They also confirm that in consideration of their intrinsically low NKT cell numbers, middle and older aged cancer patients could benefit greatly from therapeutic manipulations that boost their NKT cell numbers.

The magnitude of NKT cell activation and expansion may have critical consequences on the efficacy of NKT cell therapies. We and others have shown that NKT cells of cancer patients can be dramatically expanded by *α*-GalCer stimulation, however to levels lower that that of healthy donors (Tahir *et al*, 2001). Studies of melanoma and lung cancer patients have in contrast reported NKT cells with comparatively normal capacities to expand (Kawano *et al*, 1999a; Motohashi *et al*, 2002). As we could not assess *in vitro* NKT expansion on every cancer subject involved, we cannot determine whether NKT cell expansion is reduced in every cancer type, or whether the overall result is influenced by one or several subgroups of cancer patients.

Mechanisms of reduced responsiveness of NKT cells to *α*-GalCer stimulation have not been determined. Immune defects and dysfunction in T cells and the antigen presenting and stimulatory capacity of DC have previously been reported in cancer patients (Mizoguchi *et al*, 1992; Goto *et al*, 1999; Kiessling *et al*, 1999; Ninomiya *et al*, 1999). As *in vitro* expansion capacity is dependent upon both the number and stimulatory capacity of CDld antigen-presenting cells, reduced numbers of and/or functionally defective CDld APC may suppress NKT activation in cancer patients. Production of immunosuppressive factors by tumour cells or TGF-*β* by CD3^+^ T cells or intrinsic defects to the NKT cells themselves may also inhibit their expansion potential (Tada *et al*, 1991; Chouaib *et al*, 1997).

While functional impairment as a consequence of age has been reported to occur in other cell types such as T cells (Hakim *et al*, 2004; Plackett *et al*, 2004), there appears no association between the functional capacity of an NKT cell to respond to *α*-GalCer and the age of the healthy donor. Although fold expansion significantly decreased with age in cancer patients, this result was dependent upon three individuals with particularly high expansion. Confirmation of this latter observation would require the inclusion of an increased numbers of younger cancer patients in the subject group.

The precise relationship, if any, between the proportion of NKT cells in a donor and the NKT cell's functional capacity to expand has to date not been examined. While we have shown that there is no association between the percentage of NKT cells and fold expansion in normal donors, it is clearly evident that the largest degree of expansion was in donors with an NKT cell % at the lower end of the scale. Although fold expansion significantly increased with NKT % in cancer patients, the highly skewed nature of the population towards low NKT % makes the spread of this variable too narrow to accurately determine the correlation. NKT cells capable of dramatic expansion, some that exceeded values seen in normal donors were also prevalent within the cancer patient group. These data go a significant way to disproving the assumption held by some that people with low NKT cells will have NKT cells defective for *in vitro* expansion potential. It will be interesting to determine through clinical trials whether this translates to *in vivo* NKT cell expansion and whether this group of patients would benefit most from clinical interventions aimed at the *in vivo* activation and expansion of NKT cells.

In summary, our study demonstrates that NKT cell numbers and their activation and capacity can be modulated by both age and malignancy and in contrast to previous studies, conventional cancer treatment. Whilst further studies are required to determine both the stage at which treatment or malignant disease leads to decreased NKT cell numbers and the mechanism by which cancer affects impacts NKT cell function, our results provide important information for the clinical trials involving *in vivo* activation or large-scale *in vitro* expansion of NKT cells.
